# Can multiple miniplates improve the treatment of comminuted patellar fracture?

**DOI:** 10.1186/s12891-023-07045-x

**Published:** 2023-12-02

**Authors:** Seung Jin Yoo, Seungwoo Ok, Jaeryun Lee, Sungwook Choi

**Affiliations:** 1 Department of Orthopaedic Surgery, Jeju National University Hospital, Jeju, Republic of Korea; 2 Department of Orthopaedic Surgery, School of Medicine, Jeju National University, Jeju, Republic of Korea

**Keywords:** Comminuted patella fracture, Locking compression miniplates

## Abstract

**Background:**

We have applied primarily multiple locking compression miniplates in treating multifragmentary, comminuted patellar fracture in combination with conventional fixation methods.

**Methods:**

Medical and radiologic data were retrospectively reviewed for the patients surgically fixated with locking compression miniplates in patellar fracture of AO/OTA 34-C3. The primary outcome was bone union at the final follow-up, and the secondary outcomes were functional outcomes and postoperative complications associated with the procedure. For the functional assessment, the Lysholm score, Tegner scores, and the knee range of motion was compared.

**Results:**

A total of twenty patients with AO/OTA 34-C3 patellar fracture were included in the study with an average follow-up period of 15 months (range:11 ~ 18 months) between June 2018 and November 2021. Eleven male and nine female patients presented an average age of 57.15 years. The primary fracture union was seen in all twenty patients, and the average time to the union was 15.6 weeks on serial radiograph follow-up. All patients did not show any postoperative complications, such as fixation failure, infection, or revision operations. Postoperatively, all patients achieved an average range of motion of 130 degrees, and the Lysholm and Tegners scores showed an average of 90.4 and 5.0 at the final follow-up, retrospectively.

**Conclusion:**

Fixations with miniplates in comminuted patellar fractures can be a useful option for effective osteosynthesis due to their versatile, efficient, and low-profile nature.

## Introduction

The prevalence rate of patellar fracture is approximately 1% of all musculoskeletal trauma and multifragmentary fractures with comminution account for half of all patellar fractures [1]. Regarding the nature of intraarticular involvement in patellar fracture and the functional importance of the patella in the knee range of motion (ROM), a patellar fracture requires restoration of the extensor mechanism and accurate anatomic reduction of not only fracture fragments but also articular surface with a stable fixation for early ROM and satisfactory functional outcomes [2]. Based on the understanding of patellar fracture, the operative indications include fracture displacement greater than 3 mm, articular step-off greater than 2 mm, or disrupted extensor mechanism [3].

The majority of patellar fractures are split, transverse fractures which are amenable to anterior tension band wiring in combination with Kirschner wires or partially threaded cannulated screws [4]. However, due to a variety of patellar fracture patterns, there has not been a widely accepted gold standard surgical technique for the comminuted fracture pattern. Surgical treatment options include partial or total patellectomy, cerclage wiring, tension wiring, screw fixation, locked plating, or a combination of those [5, 6]. Each of these fixation techniques has its drawbacks. Patellectomy may result in post-traumatic arthritis, patella baja, and a decrease in the mechanical lever arm of the quadriceps, causing quadriceps weakness and extension lag [7]. Cerclage wiring may not be sufficient enough for stable fixation to allow early ROM [8] 60% of anterior tension band wiring was reported to result in symptomatic implants and postoperative knee discomfort despite a relatively low rate of fixation failures in addition to wire breakage [1, 9, 10].

Attempts to address these challenges associated with operative fixation of patellar fracture have led to the development of novel fixation techniques with conventional devices. Several plating constructs, such as a mesh plate and a fixed-angle patellar plate, have been introduced with satisfactory biomechanical outcomes [5, 6, 11]. However, these interfragmentary plating system is not readily available in all surgical settings. We have developed a novel surgical technique using locking compression miniplates in a combination of long locking screws or cannulated screws in the treatment of multifragmentary, comminuted patellar fracture. The purpose of the current case series is to evaluate and confirm the effectiveness of internal fixation with miniplates in comminuted patellar fractures, as measured by radiographic and functional outcomes.

## Materials and methods

### Patient selection

We retrospectively reviewed the medical and radiologic records of patients who were surgically treated with locking compression miniplates for patellar fractures from June 2018 to November 2021 and were followed up for at least 1 year and 3 months. This study was approved by the Institutional Review Board at our institution and was conducted under the Declaration of Helsinki.

All patients were initially evaluated by anteroposterior (AP), lateral, and Skyline radiographs of the injured knee. Patellar fractures were categorized based on the Orthopedic Trauma Association Classification (AO/OTA) scheme of the initial AP plain radiograph of the patella, along with the 3D-reconstructed computerized tomography (CT) scan taken for the understanding of the fracture anatomy and preoperative planning. The current study only included AO/OTA 34-C3 patellar fractures with more than three displaced fracture fragments and at least 3 mm of displacement and 2 mm of articular step-off. Skeletally immature patients (8 cases), patients with bipartite or tripartite patella (2 cases), and follow-up loss (3 cases) were excluded. Retrospective record reviews yielded a total of 98 AO/OTA 34 patellar fractures, and only 20 patients with AO/OTA 34-C3 multifragmentary patellar fractures were finally included and treated using 2.4 mm LCP Compact Hand miniplate-based internal fixation (DePuy-Synthes®. Warsaw, IN). All surgical procedures were performed by a single orthopedic trauma-specialized surgeon (S.C.) at our hospital trauma center.

### Clinical and radiologic evaluation

Demographics, underlying comorbidities, time to operation, operative time, postoperative complications, and clinical outcomes were assessed by reviewing admission and outpatient medical records. Time to osseous union was evaluated by periodic radiologic assessment. All patients underwent follow-up at regular postoperative intervals (1, 3, 6, 12, and last f/u) for an average of 15 months. Simple radiographs (AP, lateral) were evaluated in the immediate postoperative period and at each follow-up outpatient visit. Both the immediate postoperative and the final follow-up radiographs were compared to evaluate the accuracy of reduction and final displacement.

The primary outcome measure was osseous union, defined as the loss of fracture line and the presence of bony trabecular continuity on simple radiographs. Functional scores, using the Lysholm scores system and knee ROM, and complications associated with the procedures were assessed as secondary outcome measures from preoperative period to serial postoperative follow-ups. Potential postoperative complications include symptomatic implants, malunion, nonunion, surgical site infections, loosening of fixation devices, and re-fracture.

### Surgical protocol

Under spinal anesthesia, patients were placed in a supine position with full extension of the knee. A longitudinal midline incision over the patella was made with full-thickness skin flaps. Even though retinacula were usually disrupted by the injury, retinacula were carefully preserved and attached to the comminuted fracture fragments. The main fracture site and multi-fractured fragments were identified, and interposed hematoma and periosteum were evacuated and excised.

**Fig. 1:**
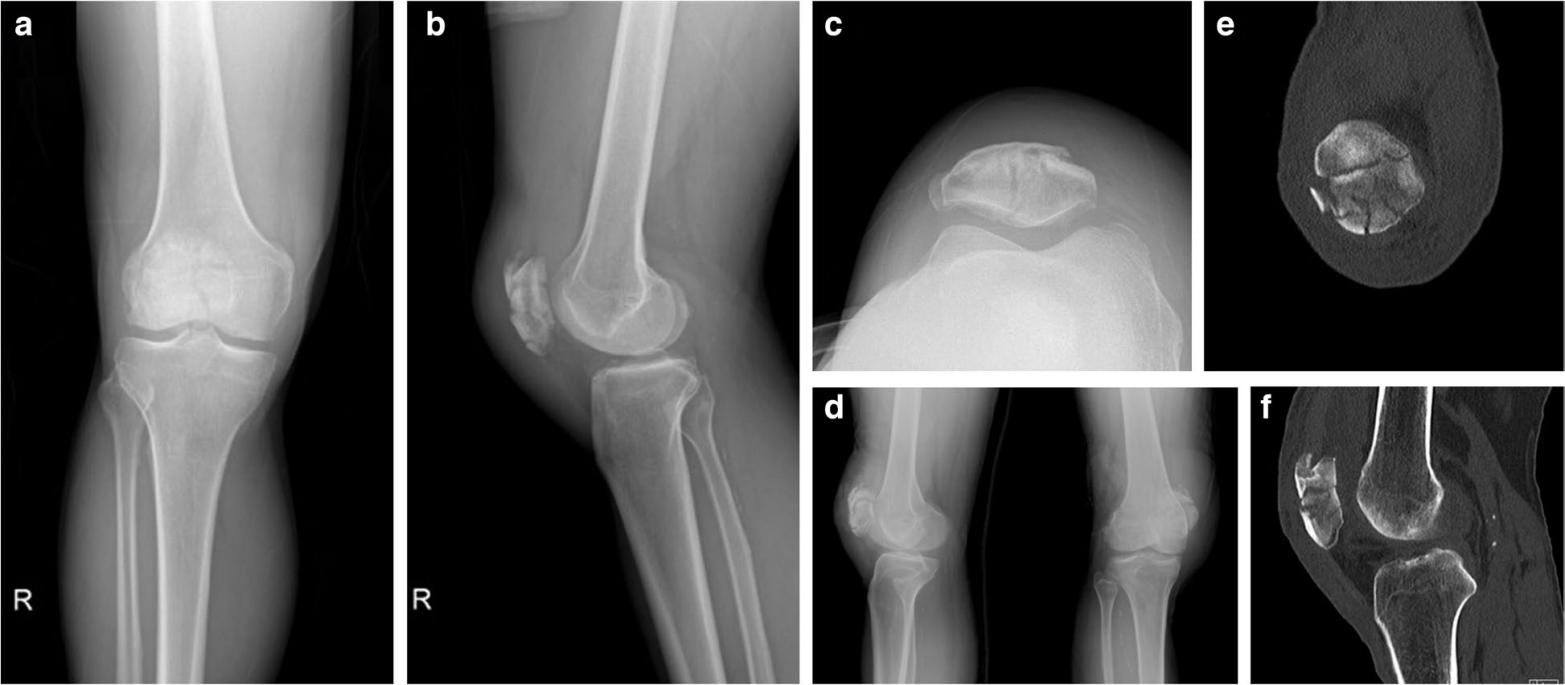
Preoperative radiologic examination of Case 1 (Male, 62 years old) **a** anterior-to-posterior (AP) view **b** lateral view **c** merchant view **d** both oblique view **e** computed tomographic (CT) coronal view **f** CT sagittal view

**Fig. 2:**
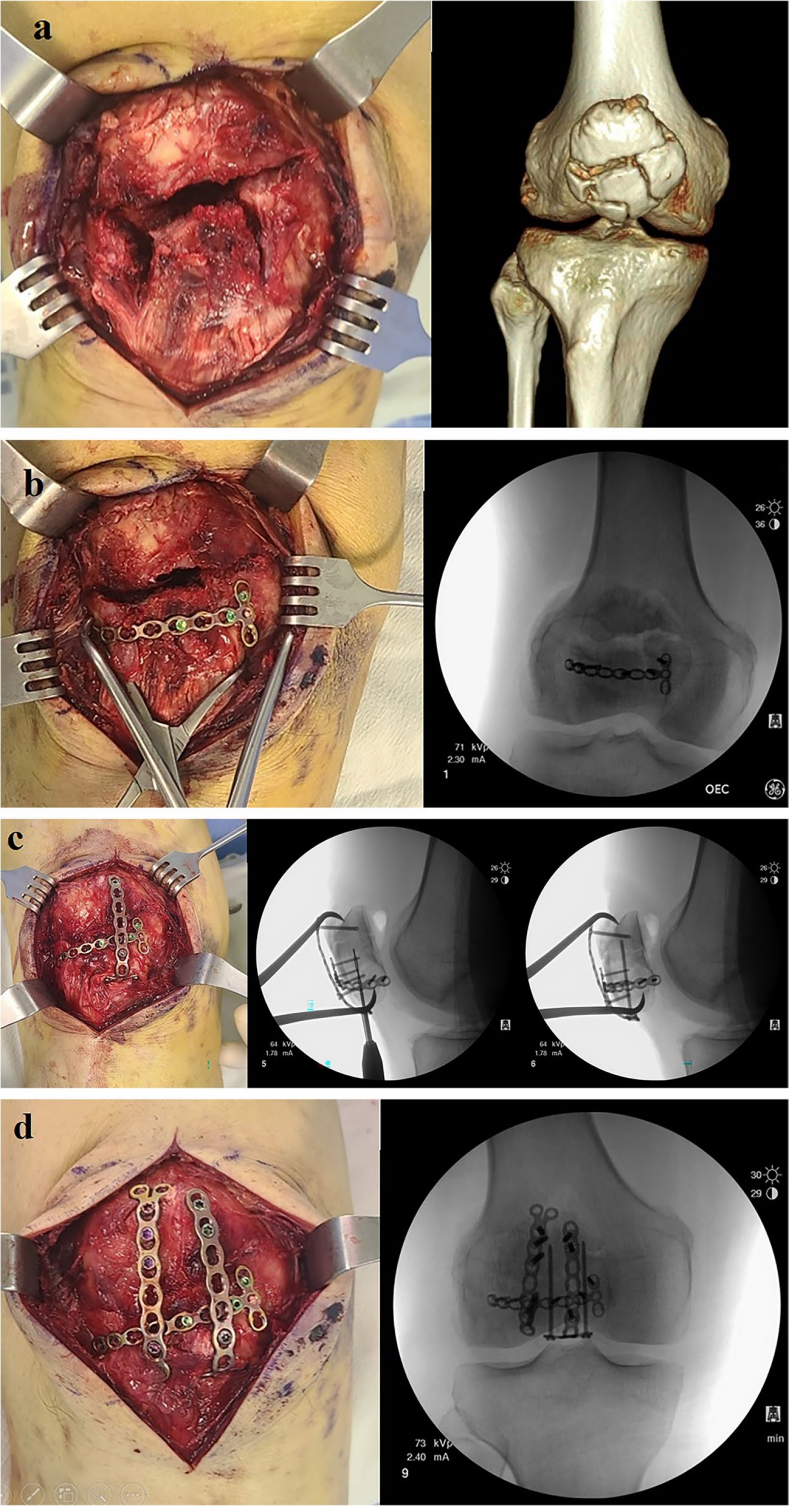
Intraoperative clinical images in correlation with C-arm fluoroscopic images of Case 1 **a** comminuted fracture pattern **b** conversion of AO classification 34-C3 to 34-C1 by reducing and fixating the inferior three fragments **c** inferiorly bent T-shaped miniplates with longitudinally inserted locking screws for better stability and compression of fragments **d** additional vertical application of miniplate for the complete transverse midbody fracture site

**Fig. 3:**
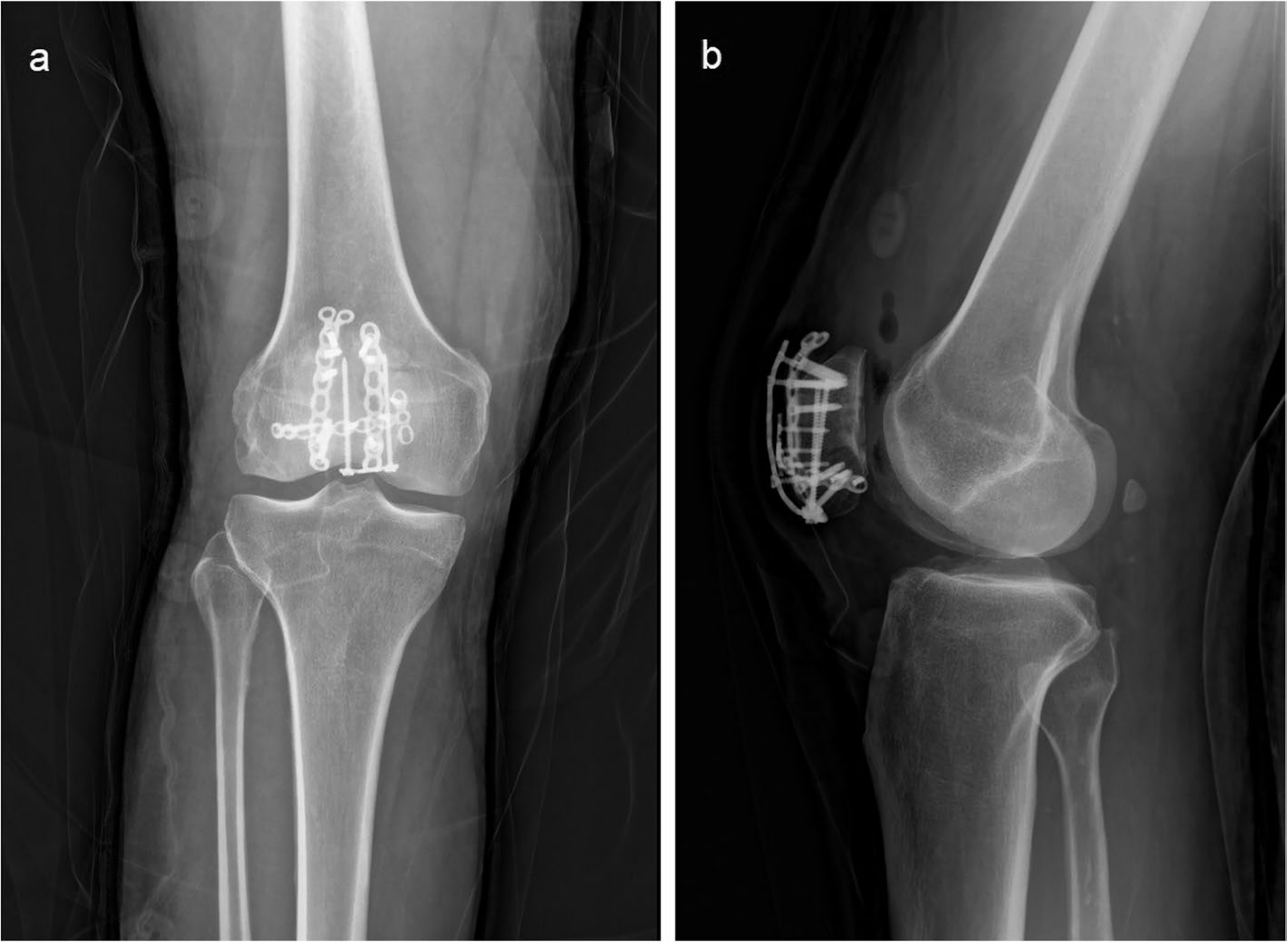
Postoperative simple radiograph of Case 1 **a** AP view **b** lateral view

Low profile 2.4 mm LCP Compact Hand miniplates were trimmed for desired lengths and angled to contour the patellar anterior osseous curvature, using cutting pliers and bending pins, respectively, to first fixate the main largest fracture fragments in the full extension of the knee. Comminuted fracture fragments were stabilized with multiple interfragmentary fixations with pre-trimmed and pre-contoured straight, Y-shaped, or T-shaped miniplates, using either cortical or locking screws. In the process of screw fixation, a depth gauge was used to assess the actual depth and to confirm no penetration into the patellar articular surface. The advantages of LCP Compact Hand miniplates and locking screws were to provide angular stability as well as to prevent screw pull-out. When necessary, long-locking screws or cannulated screws were used in combination with miniplates for supplementary stabilities (Figs. 1, 2 and 3).

#### Postoperative rehabilitation

After completing the fracture fixation, the accuracy of reduction and security of fracture gaps were examined with passive knee ROM up to 130 degrees under C-arm intensified fluoroscopy. The patients were stabilized with a soft cylinder splint postoperatively. After evaluation of knee swelling, a cylinder cast was applied in full extension with a window on the patellar area for wound management on the postoperative 2nd or 3rd day, and the patients were allowed for full weight bearing. At the postoperative 2nd week, a cylinder cast is switched to a hinged knee brace allowing the knee flexion up to 30 degrees. The patients were educated to gradually increase the allowance for the knee flexion of the hinged knee brace up to 90 degrees by the postoperative 4th week and to 130 degrees by the postoperative 6th week. The entire rehabilitation process was evaluated and modified through outpatient clinic follow-ups by the operating surgeon.

## Results

A total of 20 patients with AO/OTA 23-C3 patellar fracture were followed up for an average of 18 months (range: 14 ~ 20). The average patient age was 57.7, and ten male and ten female patients were included. All patients presented AO-OTA 34-C3 patellar fracture, and only one patient suffered from an open patellar fracture (Gustillo-Anderson type 1). Demographics and characteristics of patellar fracture are described along with types of fixations in Table 1.

**Table 1:** Demographic and clinical characteristics of patellar fractures

		20 patients
Patients		
	Age	57.7 (28~82)
	Sex	
	Male	10 (50%)
	Female	10 (50%)
Fracture Pattern (%)	AO-OTA 34-C3	20 (100%)
Affected sides	Right	12 (60%)
	Left	8 (40%)
Number of fragments (range)		4.7 (4 ~ 7)
Open Fracture (%)		1 (5%)
Fixation methods		
	Average number of miniplates per case	2.8 (2 ~ 5)
	Number of wiring (%)	5 (25%)
	Number of cerclage wiring (%)	2 (10%)
Mode of injury (%)		
	Slip down	14 (70%)
	Fall	1 (5%)
	Traffic accidents	5 (25%)
Underlying diseases (%)		
	Osteoporosis	3 (15%)
	Diabetes mellitus	5 (25%)
	Smoking	5(25%)

An average of 2.8 miniplates were used in each of the twenty cases (range, 2–5 plates per case) according to the fracture patterns. Among the twenty cases, other fixation methods used in combination with miniplates were cannulated screws (9 cases), cerclage (2 cases), and wiring (5 cases). The mean operation time was 98 min (range 59 ~ 139).

The mean follow-up periods were 18 months (range 14 ~ 20). Primary fracture union healing was seen in 20 of 20 patients (100%). The average time to bone union was 15.6 weeks (range 10 ~ 40), and the one case with anterior angulation showed delayed bone union at the postoperative 40th week. There was no fixation failure or postoperative infection. At the final follow-up, seven patients underwent implant removal at patients’ will, but no patients complained of symptomatic implants. All of the patients achieved the postoperative average ROM of 130 degrees by the postoperative 3rd month, and the mean Lysholm score at the final follow-up was 90.4 (Table 2).

**Table 2:** Radiologic and clinical outcomes

	20 patients
Primary union rates (%)	20 (100%)
Union times (weeks)	15.6
Operation times (minutes)	98 (59 ~ 139)
Complications (%)	
Malunion	0 (0%)
Fixation failure	0 (0%)
Postoperative infection	0 (0%)
Additional surgery (%)	
Revision surgery (%)	0 (0%)
Implant removal (%)	7 (35%)
Average final range of motion	120 (100 ~ 135)
Incidence of extension lag	0 (0%)
Average Lysholm scores	
Postoperative 1 month	44.3 (20 ~ 52)
Postoperative 3 month	58.5 (28 ~ 74)
Postoperative 6 month	89.1 (58 ~ 100)
Postoperative 12 month	90.4 (68 ~ 100)
Last follow up	92.1 (72 ~ 100)
Average Tegner scores	
Postoperative 1 month	0.8 (0 ~ 2)
Postoperative 3 month	1.9 (1 ~ 3)
Postoperative 6 month	3.6 (2 ~ 6)
Postoperative 12 month	4.3 (2 ~ 7)
Last follow up	5.0 (3 ~ 9)

## Discussion

The primary goal in the surgical treatment of the patellar fracture is to reduce the articular surface of the patella anatomically, to restore the extensor mechanism of the knee, and to provide firm fixation to allow early ROM of the knee joint for better functional recovery. However, the management of the comminuted patellar fracture is challenging with conventional fixation methods to achieve adequate multifragmentary reduction or fixation and timely movement of the knee joint. The objective of the current clinical case series was to describe a novel fixation technique primarily based on the use of widely available locking compression miniplates for hand fractures in combination with cannulated screws that can be easily adapted for comminuted patellar fractures. Unlike a few previously reported literature on the use of miniplate in patellar fracture, the current case series mainly focuses on the effectiveness of miniplate usages on the severely comminuted patellar fracture (AO-OTA 34-C3) [10, 11]. The findings of our study suggest that osteosynthesis of patella fracture with Compact hand miniplates can provide sufficient mechanical stability for early ROM without postoperative complications such as nonunion, fixation failure, and symptomatic implants.

Despite a wide array of fixation techniques, such as conventional tension band wiring with K-wires, wiring with cannulated screws, circumferential cerclage wiring, and partial or total patellectomy, complex patterns of comminuted patellar fracture lack a standard protocol for surgical management. Tension band wiring with K-wires or cannulated screws in combination with wiring often is insufficient to purchase all fractured bony fragments and to provide adequate fixation in comminuted patellar fracture, and a biomechanical study has found tension band wiring with K-wire is inferior in a load to failure than with cannulated screws [9]. With advances in locking plates, the indications for osteosynthesis with locking plates include complex periarticular fractures, comminuted metaphyseal or diaphyseal fractures, periprosthetic fractures, and fractures in osteoporotic bones [12]. Furthermore, several biomechanical studies on locking plates have demonstrated significantly increased stability toward torsional or bending loads as well as ultimate failure strength, even in patellar fracture [6, 13, 14]. Another study on the stability of the locking plate at a fracture site reported that the longer length of the plate to span the fractured segments, the smaller pullout forces acting on the screw-bone interface, and the lower levels of strain at the fracture site and plates [15]. In addition, a biomechanical cadaveric study comparing fixed-angle plates to conventional tension band wiring and cannulated screws with additional anterior tension wiring in patellar fracture showed that plate fixation has yielded lower mean tensile load at the fracture site and less fracture gap widening after repetitive cyclic loads than other fixation methods [4]. In addition, osteosynthesis with low-profile plates in patellar fracture has been considered an alternative fixation method to achieve satisfactory union rates with significantly reduced incidences of symptomatic hardware [11, 16, 17]. Anatomically contoured patellar locking plate also showed satisfactory outcomes in postoperative range of motion, patients’ satisfaction, and complication rates [18].

Based on the biomechanical and clinical advantages of plate osteosynthesis in patellar fracture evidenced in the previous literature, we have developed a novel surgical technique using locking compression miniplates in combination with cannulated screws. There have been a few previous studies on patella-specific plates, such as anatomically shaped patellar locking plates, bilateral fixed angle basket plates, or titanium mesh plates, but those types of plates are not readily available in all surgical settings [6, 16, 19].

In the current study, we utilized 2.4 mm LCP Compact Hand miniplate-based internal fixation (DePuy-Synthes®. Warsaw, IN) that is widely used in hand fractures; furthermore, these miniplates are advantageous in its low-profile construct and versatility in multiplanar application. Because anatomic reduction under direct visualization of complex fracture pattern in a full extension of the knee was performed with multiplanar and low-profile miniplates in combination with locking screws or cannulated screws, it was easier to manipulate multifragmentary fracture and was able to prevent extensive disruption of vascular supply or periosteum. In addition, unlike specifically designed cage patellar plates, widely available miniplates for its versatility and easy multiplanar manipulation are definite benefits in comminuted patellar fracture.

**Fig. 4:**
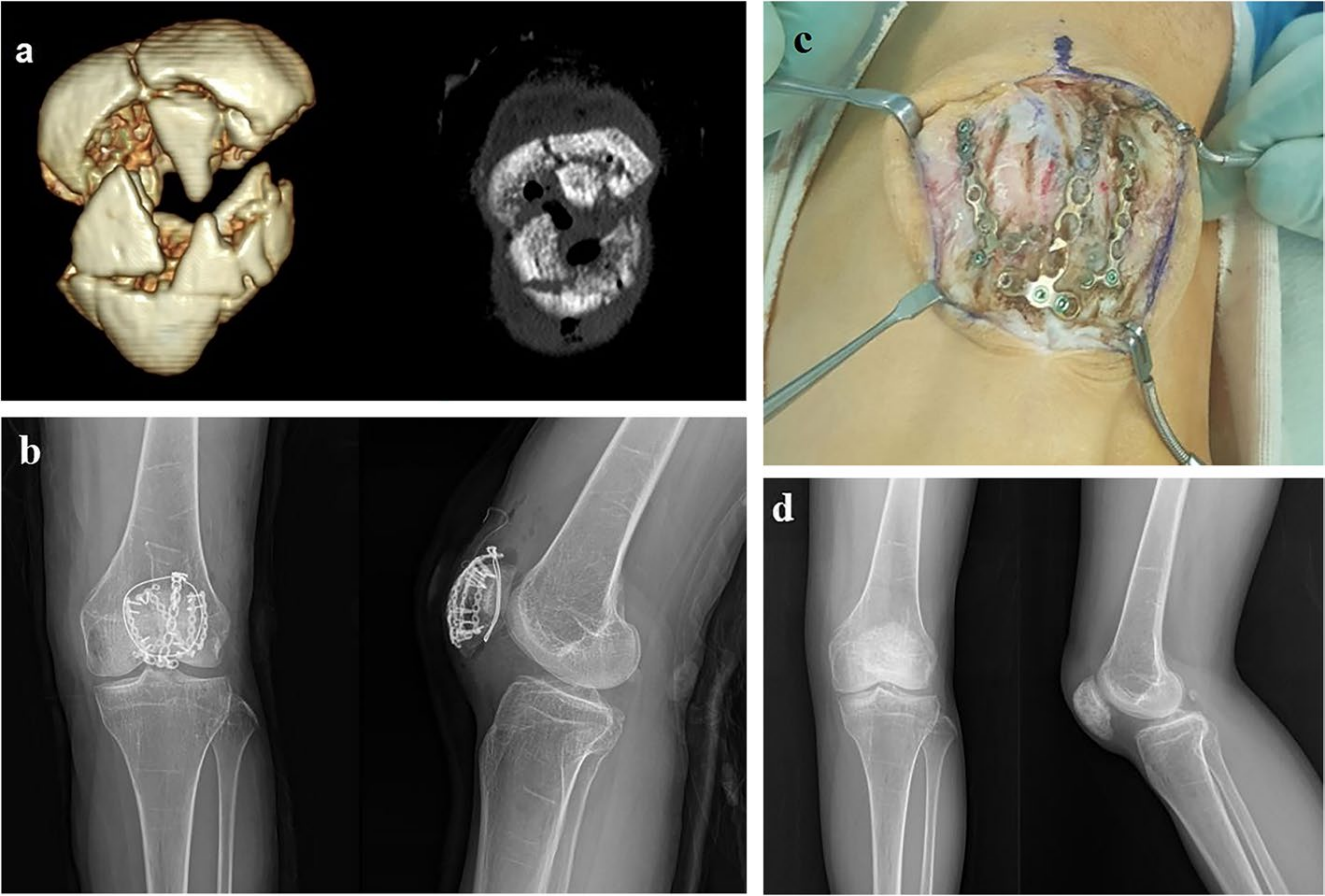
Preoperative and postoperative radiologic examination of Case 2 (Female, 52 years old) **a** preoperative CT coronal and 3D reconstructed image **b** postoperative simple radiograph AP and lateral view after internal fixation **c** intraoperative clinical photo of miniplates at the time of implant removal **d** postoperative simple radiograph AP and lateral view after implant removal

In the current cases, all comminuted patellar fractures involved the inferior pole, and the miniplate was effectively able to stabilize the inferior pole comminution [20]. Due to complex and unique fracture patterns in each case of patellar fracture, it was difficult to describe each type of plate or screws used in the current case series; however, the main purpose of miniplate application in the comminuted patellar fracture is to neutralize fractured fragments (Fig. 4). With an average of 18 months of postoperative follow-up, all patients achieved bone union and satisfactory functional outcomes without postoperative complications such as symptomatic hardware or limitation in ROM.

Our study has some limitations. The current study was retrospective in its nature, and the relatively small size of our study without a control group or comparative groups may limit the generalizability of our results. However, our novel surgical technique with widely available conventional miniplates to show satisfactory bone healing and functional outcomes is a major strength of our study. A biomechanical study on the miniplate application in comparison to other patellar plate constructs or other fixation methods would be useful.

## Conclusion

In conclusion, comminuted patellar fractures are one of the most clinically challenging orthopedic trauma. The surgical techniques herein described satisfactory clinical, radiologic, and functional evidence for treating difficult, comminuted patellar fracture with conventional Compact hand-locking compression miniplates.

## Data Availability

The dataset used and/or analyzed during the current study available from the corresponding author on reasonable request
